# Effectiveness of EV-A71 Vaccine and Its Impact on the Incidence of Hand, Foot and Mouth Disease: A Systematic Review

**DOI:** 10.3390/vaccines12091028

**Published:** 2024-09-08

**Authors:** Quanman Hu, Yaqi Xie, Fucang Ji, Fei Zhao, Xiaoru Song, Saiwei Lu, Zijie Li, Juan Geng, Haiyan Yang, Jinzhao Long, Yuefei Jin, Shuaiyin Chen, Guangcai Duan

**Affiliations:** 1 College of Public Health, Zhengzhou University, Zhengzhou 450001, Chinagcduan@zzu.edu.cn (G.D.); 2 Center for Disease Control and Prevention of Zhengyang, Zhengyang, Zhumadian 463600, China

**Keywords:** EV-A71 vaccine, effectiveness, meta-analysis, test-negative design, HFMD, epidemiological characteristics

## Abstract

Background: Vaccination is a highly effective strategy for the prevention of enterovirus A71 (EV-A71)—hand, foot, and mouth disease (HFMD). Three inactivated EV-A71 vaccines in China have demonstrated remarkable efficacy against EV-A71-HFMD during clinical trials, exhibiting vaccine effectiveness (VE) exceeding 90% and few adverse events (AEs). However, the effectiveness of vaccines in the real world and its impact on the epidemiological characteristics of HFMD after the use of EV-A71 inactivated vaccine are uncertain. Methods: The odd ratio (OR) and 95% confidence (CI) were used as the effect estimates of the meta-analysis in the test-negative design (TND), and the OR was used to calculate VE: VE = (1 − OR) × 100%. Results: According to the literature search strategy, a comprehensive search was conducted in PubMed, Web of Science (including Chinese Science Citation Database and MEDLINE), and Embase, and 18 records were ultimately included in this study. Subsequently, the overall VE and 95% CI of different vaccine doses were analyzed, with the one-dose vaccine at 66.9% (95% CI: 45.2–80.0%) and the two-dose vaccine at 84.2% (95% CI: 79.4–87.9%). Additionally, the most reported AEs were mild general reactions without any rare occurrences. Simultaneously, the widespread use of the EV-A71 vaccine would lead to a reduction in both the incidence of EV-A71-associated HFMD and severe cases caused by EV-A71. Conclusion: The administration of the two-dose EV-A71 vaccine is highly effective in preventing HFMD in the real world, and the widespread use of the EV-A71 vaccine leads to a reduction in the incidence of EV-A71-associated HFMD and that of severe cases caused by EV-A71. The findings suggest that administering the two-dose EV-A71 inactivated vaccine to children aged 6 months to 71 months can be effective in preventing EV-A71-associated HFMD, highlighting the need for developing a multivalent HFMD vaccine for preventing cases not caused by EV-A71.

## 1. Introduction

Hand, foot, and mouth disease (HFMD) is a prevalent contagious illness that mainly impacts children aged 5 and younger. The primary causative pathogens are enterovirus A71 (EV-A71), coxsackievirus A16 (CVA16), and CVA6 [1,2]. Although the majority of HFMD cases exhibit mild symptoms, acknowledging that a minority of children may experience severe manifestations, including encephalitis, pulmonary edema, and even fatality, is important [3,4]. Studies have demonstrated the neurotropism of EV-A71, which can interact with human scavenger receptor B2, P-selectin glycoprotein receptor 1, and other receptors upon infection to invade the nervous system [5]. In children, this invasion leads to central nervous system damage, encephalitis, neuronal degeneration, and related disorders, triggering a cascade of inflammatory responses in conjunction with lung mast cells and the renin–angiotensin system. Consequently, acute lung injury ensues along with circulatory system failure and multiple organ dysfunction syndrome within a short timeframe. This condition exhibits high mortality rates and poor prognosis [6,7]. In 2008, Fuyang, China experienced a severe epidemic outbreak of HFMD, resulting in 488,955 reported cases nationwide that year alone. EV-A71 is the major pathogen responsible for severe cases of HFMD and accounts for 93% of HFMD-related fatalities. Given its remarkable impact on child health, it was classified as a Class C infectious disease by Chinese authorities and has since emerged as an important public health concern [8,9]. However, effective antiviral therapies targeting EV-A71 infection are currently unavailable.

Therefore, vaccination may be a highly effective strategy for EV-A71-HFMD prevention. In December 2015, the China Food and Drug Administration approved inactivated vaccines specifically designed to combat EV-A71-HFMD. Currently, three vaccines are commercially available (including Institute of Medical Biology, Chinese Academy of Medical Science; Sinovac Biotech Ltd.; Beijing Vigoo Biological Co., Ltd.). They demonstrated remarkable efficacy against EV-A71-HFMD during clinical trials, exhibiting vaccine effectiveness (VE) exceeding 90% and few adverse events (AEs) [10,11,12]. Moreover, the Chinese Center for Disease Control and Prevention has issued technical guidelines for the utilization of the EV-A71 inactivated vaccine, recommending self-financed vaccination. The target population for EV-A71 vaccination includes susceptible children aged ≥6 months, with an emphasis on early administration for optimal efficacy. Encouragement is given to complete the vaccination regimen before 12 months of age to ensure early protection [13]. However, the effectiveness of the inactivated EV-A71 vaccine remains uncertain in the real word. The effectiveness of numerous existing vaccines, such as the influenza vaccine [14], COVID-19 vaccine [15], and the rotavirus vaccine [16], in the real world is evaluated by employing a test-negative design (TND). As a type of case–control study, the TND study exhibits fewer biases than other observational studies [17,18]. TND was employed to assess the effectiveness of the EV-A71 inactivated vaccine through standardized laboratory tests for viral infection. The subjects were segregated into positive and negative groups, and the VE rates of the two groups were compared.

However, the TND studies on the effectiveness of EV-A71 inactivated vaccine exhibit considerable heterogeneity; for example, Duan et al. reported the VE for the two-dose vaccine to be 63.4% (35.2–79.4%) [19], whereas Jiang et al. demonstrated a high VE of 88.3% (67.7–95.8%) for the same two-dose vaccine [20]. Additionally, the impact of the mass administration of the EV-A71 inactivated vaccine on the prevalence, pathogen composition, severity rate, and age distribution of HFMD remains unknown, and varying methodologies have been employed across different studies. Therefore, this systematic review aims to provide robust evidence-based support for the effectiveness of vaccines in real-world vaccination scenarios and summarize the impact on the epidemiological characteristics of HFMD after the use of the EV-A71 inactivated vaccine. Thus, it may offer valuable insights for the future development of HFMD-related vaccines.

## 2. Materials and Methods

### 2.1. Literature Search Strategy

A comprehensive search was conducted through PubMed, Web of Science (including Chinese Science Citation Database, MEDLINE), and Embase for published literature from 1 January 2016 to 21 April 2024. The terms used for the search were (“hand-foot-mouth disease” or “hand foot mouth disease” or “hand, foot and mouth disease” or “HFMD”), (“Enterovirus A71” or EV-A71 or EV71), and (vaccine or vaccination).

The articles included in this study underwent a thorough search for relevant reviews and meta-analyses, which confirmed that no identical publications were found. Subsequently, the titles and abstracts of the identified articles underwent screening, followed by downloading of pertinent papers.

### 2.2. The Criteria for Inclusion and Exclusion

The criteria for inclusion were as follows: (1) studies investigating the VE or AEs of EV-A71 vaccine in the actual inoculation environment and (2) studies describing the epidemiological characteristics of HFMD after the use of EV-A71 inactivated vaccine. Screened records meeting any of the criteria were included in the study.

The criteria for exclusion were as follows: (1) reviews, erratum and comments; (2) studies that focus only on a specific population, such as children aged six months; and (3) studies that include EV-A71 in combination with other vaccines and co-vaccination.

### 2.3. Literature Screening and Data Extraction

This study was carried out in full compliance with the PRISMA protocol 2020 [21].

According to the predefined inclusion and exclusion criteria, two authors independently screened the literature retrieved from the searched database. In case of any disagreement, a third author was consulted for resolution. Subsequently, data extraction was performed encompassing key information such as first author, study design, area, study type, period, age r, and male/female. The Newcastle–Ottawa scale (NOS) was used to evaluate the quality of the TND studies: NOS score ≥ 7 is high quality, 5 ~ <7 is moderate quality, and <5 is low quality [22].

### 2.4. Statistical Analysis

The statistical analyses were conducted using the software STATA version 12.1 software (Stata Corp, College Station, TX, USA), with a significance level set at *α* = 0.05. The odd ratio (OR) and 95% confidence (CI) were used as effect estimates, and OR was used to calculate VE: VE = (1 − OR) × 100%. The I^2^ statistic was employed to measure the heterogeneity among the studies. I^2^ > 50% or *p* < 0.05 suggested significant statistical heterogeneity. In this case, a random effects model was used considering the intra- and inter-study variation. Otherwise, the pooled effect was calculated using a fixed-effects model. Subgroup analysis was used to investigate the potential origin of heterogeneity and relied on severity, age period, and other relevant variables. Publication bias was assessed using funnel plot and Egger’s test. Sensitivity analysis was used to assess whether the study results were reliable [23,24].

## 3. Results

### 3.1. Study Selection and the Records of Included Studies

**Figure 1:**
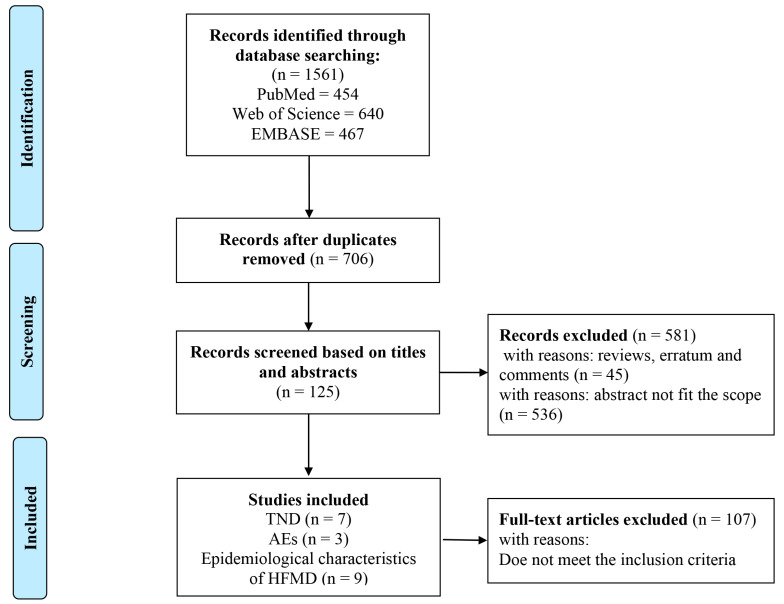
Flowchart showing the screening process for included articles.

According to the literature search strategy, a comprehensive search was conducted in PubMed, Web of Science (including Chinese Science Citation Database and MEDLINE), and Embase. As shown in Figure 1, a total of 1561 records were retrieved. A total of 125 records remained after the duplicates were removed and the title and abstract were read. According to an assessment of the full texts, seven records were about VE [19,20,25,26,27,28,29], three records were about AEs [26,30,31], and nine records were about the epidemiological characteristics of HFMD after the introduction of the EV-A71 inactivated vaccine [32,33,34,35,36,37,38,39,40].

### 3.2. Overall VE/OR and 95% CI

#### 3.2.1. The Characteristics of Included Studies about VE

Table 1 shows that among the included studies, three were prospective and four were retrospective, with the study period concentrated between 2017 and 2019. The study objects of the five studies were aged 6 to 60/71 months, whereas those of the two studies were aged from 6 months to 35 months. All studies had a sample size exceeding 1000 participants, with a maximum reaching 18,860 individuals. Moreover, the male/female ratio in these studies exceeded 1 (1.27–1.98). The NOS score of all studies was 7 or higher, with a maximum of 9. The details are shown in Table S2.

#### 3.2.2. Overall VE/OR and 95% CI

**Figure 2:**
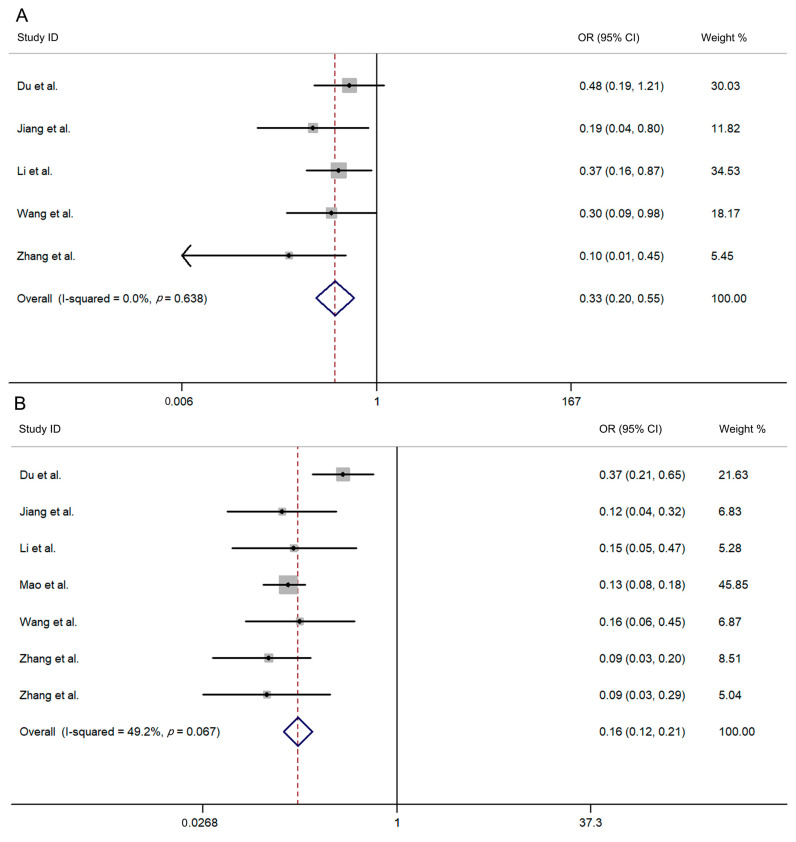
Forest plot showing the overall OR and 95% CI of different vaccine doses. (**A**) one dose; (**B**) two dose [19,20,25,26,27,28,29].

We analyzed the overall VE/OR and 95% CI of different vaccine doses: The overall VE of the one-dose vaccine is 66.9% (95% CI: 45.2–80.0%), and the overall OR of the one-dose vaccine is 0.33 (95% CI: 0.200–0.548, I^2^ = 0.0%, *p* = 0.638; Figure 2A). The overall VE of two-dose vaccine is 84.2% (95% CI: 79.4–87.9%), and the overall OR of two-dose vaccine is 0.158 (95% CI: 0.121–0.206, I^2^ = 49.0%, *p* = 0.067; Figure 2B).

#### 3.2.3. Subgroup Analysis

**Figure 3:**
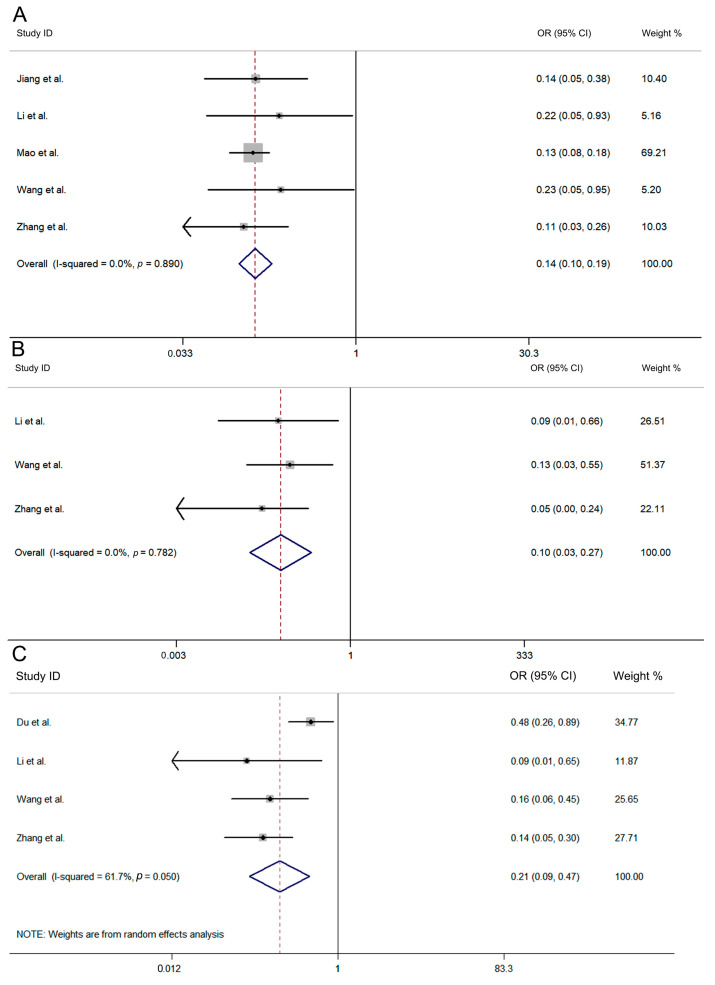
Subgroup analysis was conducted on children aged 6–35 months (**A**); children aged 36–71 months (**B**); clinical severity = no severe disease progression (**C**) [19,20,25,26,27,28,29].

The subgroup analysis was conducted on children who received the two-dose vaccine. The results revealed that the VE for children aged 6–35 months was 86.3% (95% CI: 81.0–90.1%) and the OR was 0.137 (95% CI: 0.099–0.190, I^2^ = 0.0%, *p* = 0.890, Figure 3A), whereas that for children aged 36–71 months was 90.3% (95% CI: 72.8–96.6%), with an OR of 0.097 (95% CI: 0.034–0.272, I^2^ = 0.0%, *p* = 0.782; Figure 3B). The VE for children with no severe disease progression was 78.9% (95% CI: 52.8–90.6%), with an OR (95% CI: 0.094–0.472, I^2^ = 61.7%, *p* = 0.050; Figure 3C). Among the studies where disease progression was severe, three reported a VE of 100% (95% CI: 100–100%) [14,22,23], whereas the VE of another study reached 88.3% (95% CI: 67.7–95.8%) [15].

#### 3.2.4. Publication Bias and Sensitivity Analysis

**Figure 4:**
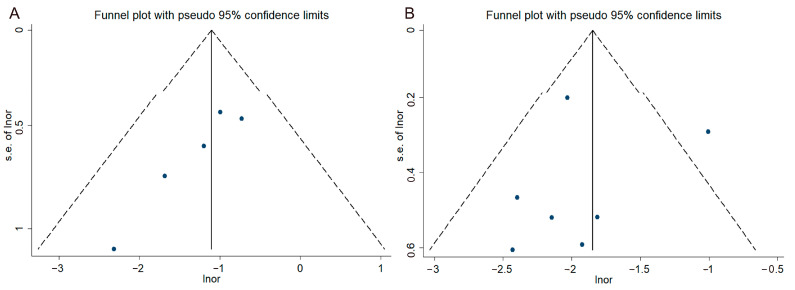
Funnel plot on different vaccine dose. (**A**) one dose; (**B**) two dose.

**Figure 5:**
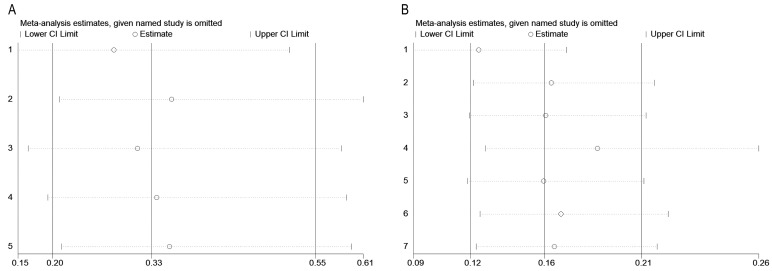
Sensitivity analysis on different vaccine dose. (**A**) one dose; (**B**) two dose.

The publication bias was assessed using a funnel plot and Begg’s test, as shown in Figure 4A,B. No significant publication bias was detected (z = 0.00, *p* = 1.00) for the one-dose vaccines and the two-dose vaccine (Z = 1.71, *p* = 0.086). Additionally, the sensitivity analysis results demonstrated the stability and reliability of our findings (Figure 5A,B).

### 3.3. Adverse Events

The AEs following vaccination with the EV-A71 vaccine remain unknown, but there are insufficient studies of relevance to facilitate a meta-analysis. Table 2 shows that Mao et al. reported an incidence rate of 1.29‰ for AEs in children aged 6–35 months post-vaccination, with local and systemic AEs occurring at rates of 0.95‰ and 0.88‰, respectively; local and systemic AEs are predominantly classified as grade I and II AEs, respectively, without any grade IV reactions observed [26]. Furthermore, variations in AEs were noted across different vaccine doses. Within 3 days of inoculation, the one-dose vaccine exhibited an AE rate of 2.045%, whereas the two-dose vaccine had a rate of 1.611%. Systemic AEs were predominant, whereas local AEs were infrequent [30]. Additionally, Luo et al. synthesized the data on AEs within the time intervals of 30 min, 3 days, and 4–30 days after vaccination, with incidence rates of 0.33%, 1.58%, and 0.34%, respectively. Most studies reported that the AEs were mild general reactions without any rare occurrences. [31].

### 3.4. Epidemiological Characteristics of HFMD after the Use of EV-A71 Inactivated Vaccine

The impact of the EV-A71 inactivated vaccine on the prevalence of HFMD remains uncertain. Table 3 shows that Duan et al. conducted a comprehensive virological surveillance of HFMD in Chengdu over a 5-year period (2017–2022). The results showed a remarkable decline in the rate of EV-A71 infection following the introduction of the EV-A71 vaccine. This serotype is no longer dominant among the laboratory-confirmed severe HFMD cases, accounting for only 15.6% [32]. These findings are consistent with previous studies [33]. Another study also reported that EV-A71 vaccination does not reduce the overall HFMD incidence but increases it instead [34,35]. Furthermore, the comparison of pre-vaccination (2012–2016) and post-vaccination (2017–2020) HFMD epidemic data shows that the proportion of EV-A71-HFMD cases decreases from 35.37% to 4.45% [36]. Similarly, the proportion of severe HFMD cases caused by EV-A71 decreases from 62.4% to 28.5%, and a shift toward older age groups affected by EV-A71 infection exists [37]. This finding is consistent with the findings of Hong et al., indicating a decrease in proportions among children under 3 years old and an increase among those aged between 3 and 5 years old [38].

In addition, some time series models were employed to forecast the prevalence of HFMD after the administration of the EV-A71 inactivated vaccine. The Bayesian structural time series models by Wu et al. demonstrate that if no vaccination measures are implemented and the incidence of EV-A71-HFMD remains unchanged, then the anticipated incidence in this case would be 2.76 times higher than that in the case with vaccination [39]. The incorporation of vaccination as a change point model into the time series analysis results in an estimation indicating that 6911 cases of EV-A71 can be prevented over a span of 2 years; this outcome can result in a reduction in severe HFMD cases by 52% (ranging from 42% to 60%) [40].

## 4. Discussion

Large-scale clinical trials of vaccines before marketing are necessary. However, clinical trials are conducted for VE evaluation under ideal circumstances, which cannot simulate the real-world vaccination situation. In actual vaccination situations, the protective effect of vaccines may be affected by various factors, such as vaccination population, epidemic characteristics, and vaccination methods. Thus, the results of clinical trials and the results of vaccine protection have certain differences.

TND, a reliable method used in case–control studies, is frequently utilized to evaluate the effectiveness of vaccines in the real-world vaccination scenarios [41,42]. De et al. demonstrated that TND yields consistent VE estimates form clinical trials for the live attenuated human influenza vaccine [43] and rotavirus vaccine [16]. In terms of feasibility and ethical considerations, TND emerges as the preferred method for evaluating VE post-licensure of the EV-A71 vaccine. In this systematic review and meta-analysis, we used the TND methodology to evaluate the VE of the EV-A71 vaccine under actual vaccination conditions. The results of this study demonstrated remarkable differences in VE doses, with the one-dose vaccine showing a VE of 66.9% (95% CI: 45.2–80.0%) and the two-dose vaccine showing a VE of 84.2% (95% CI: 79.4–87.9%). These findings suggest that vaccines administered according to the recommended immunization schedule provide enhanced protection against EV-A71 and HFMD [13]. However, the VE of the two-dose vaccine in our review exhibits a slight decrease compared with those in Phase III and Phase IV clinical trials [44,45], potentially because of the strict regulations for participant inclusion and grouping and the comprehensive definitions of EV-A71 and HFMD employed in clinical trial settings.

We subsequently conducted a subgroup analysis based on the age and clinical severity of VE administration by using the two-dose vaccine. A positive correlation was observed between the age of children receiving the two-dose vaccine and the efficacy of vaccine protection. These findings are consistent with the findings in the Phase III clinical trial, where the geometric mean titer levels detected in the older group (36–71 months) were higher than those detected in the younger group (6–35 months) when the same vaccine was employed [46]. A similar phenomenon was observed in another Phase III clinical trial [47]. The potential explanation lies in the close association between the seropositive rate of serum anti-EV-A71 antibody and age. A seroepidemiological study conducted in Jiangsu Province, China, revealed a gradual decline in seropositivity of the anti-EV-A71 antibody from children aged 0–6 months; this gradual decline is followed by sustained low levels at 7–11 months and a subsequent increase during ages 1–4 years [48]. Furthermore, a meta-analysis also demonstrated a direct correlation between age and EV-A71 antibody seropositivity, with rates increasing from 26% at age 1 to 70% at age 5 [49]. In terms of clinical severity, the VE for HFMD cases with no severe disease progression is lower than that for cases with severe disease progress. Three included studies consistently demonstrate a 100% effectiveness of the vaccine against severe cases; this finding aligns with the findings from a multicenter clinical trial: the vaccine exhibits 100% efficacy in preventing HFMD with neurological complications (95% CI: 42.6–100%) [11].

In recent years, the widespread administration of the EV-A71 vaccine in China has resulted in remarkable shifts in the pathogen spectrum of HFMD. The rate of EV-A71 infection significantly declines, and EV-A71 is no longer dominant among laboratory-confirmed severe HFMD cases [50]. However, the EV-A71 vaccine did not reduce the incidence of HFMD in certain regions; instead, it exhibited an increase [34]. This finding can be potentially attributed to the emergence of other enteroviruses, such as CVA6 [2], CV-A16 [51], and CVA10 [52], as predominant pathogens causing local HFMD. It also highlights the remarkable variations among regions. CVA6 has emerged as the primary causative agent in various countries and regions, such as France [53] and Brazil [54]. Furthermore, Du et al. demonstrated a significant inverse correlation between the administration of the EV-A71 vaccine to 3-year-olds and the incidence of HFMD, with each 1% increase in vaccination coverage resulting in a corresponding decrease of 0.9% in HFMD cases [55]. Given factors such as environmental pollution and quarantine, mathematical models have substantiated that the implementation of vaccine measures since 2016 has reduced the total patient count by 17% and 22% in 2016 and 2017, respectively [56].

However, the EV-A71 vaccine does not confer cross-immunity against other enterovirus-associated HFMD. Currently, a bivalent inactivated EV-A71-CA16 vaccine demonstrates favorable safety profiles in mice; this result provides sufficient evidence for its potential use in clinical trials [57]. Furthermore, Liu et al. parameterized HFMD detection data using a static model and concluded that, compared with the univalent EV-A71 vaccine, the bivalent EV-A71-CA16 vaccine exhibits superior cost-effectiveness [58]. Zhang et al. also developed a quadrivalent vaccine against HFMD using recombinant virus-like particles in mice. The passively transferred immune sera from the quadrivalent vaccine demonstrate effective protection against single or mixed infections of mouse EV-A71, CVA16, CVA10, and CVA6 [59]. These findings suggest that the development and clinical trials of a multivalent HFMD vaccine represent a promising approach for preventing HFMD in the future. Furthermore, the inactivated EV-A71 vaccine has been administered concomitantly with various vaccines, such as the measles-mumps-rubella [60] and the trivalent split-virion inactivated influenza vaccine [61], exhibiting favorable immunogenicity and safety profiles in clinical trials.

This study also has several limitations: (1) The insufficient inclusion of studies and the limited number of subgroup analyses preclude the possibility of conducting a meta-analysis. (2) Variations in case selection criteria among the included TND studies are evident; some focus solely on clinically severe cases, whereas others concentrate on cases enrolled in the HFMD virological surveillance system (including mild and severe cases). (3) The quantitative analysis of the impact of EV-A71 inactivated vaccine on HFMD epidemiology is lacking. Thus, further research and quantitative analysis are necessary. (4) The evaluation of the effect of EV-A71 inactivated vaccines on non-EV-A71-HFMD was not conducted, thereby highlighting the need for a disaggregated analysis in the future.

## 5. Conclusions

In conclusion, the administration of the two-dose EV-A71 vaccine is highly effective in preventing HFMD in the real world. In particular, the VE of the one-dose vaccine is comparatively lower than that of the two-dose vaccine. Moreover, the widespread use of the EV-A71 vaccine led to a reduction in the incidence of EV-A71-associated HFMD and that of severe cases caused by EV-A71. The findings suggest that administering the two-dose EV-A71 inactivated vaccine to children aged 6 months to 71 months can be effective in preventing EV-A71-associated HFMD, highlighting the need for developing a multivalent HFMD vaccine for preventing cases not caused by EV-A71.

## Figures and Tables

**Table 1:** The characteristics of included studies about VE.

Author	Design	Vaccines	Endpoint	Population	Area	Type	Study Period	Age	Sample	Male/Female	NOS
Du et al. [19]	TND	Mixed, not clear	HFMD cases	Hospital	Sichuan, China	R	June 2017–March 2022	>6 months	4833	1.63	7
Jiang et al. [20]	TND	Mixed, not clear	HFMD severe cases	Individual records of all severe cases	Guangxi, China	R	January 2017–December 2018	6–60 months	2779	1.98	7
Li et al. [25]	TND	Mixed, not clear	HFMD cases	Hospital	Henan, China	R	February 2017–February 2018	6–71 months	1803	1.97	9
Mao et al. [26]	TND	Sinovac Biotech Ltd. and Beijing	HFMD cases	Individual records of all cases	Wenzhou, China	P	January 2019–December 2019	6–35 months	18860	1.27	7
Wang et al. [27]	TND	Mixed, not clear	HFMD cases	Individual records of all cases	Beijing, China	R	January 2017–December 2017	6–59 months	2184	1.53	8
Zhang et al. [28]	TND	Mixed, not clear	HFMD cases	Individual records of all cases	Three provinces, China	P	January 2019–December 2019	6–71 months	3223	1.48	7
Zhang et al. [29]	TND	Vigoo Biological Co., Ltd.	HFMD cases	From phase 3 trial	Jiangsu, China	P	NA	6–35 months	7325	1.30	7

Note: TND, test-negative design; R, retrospective; P, prospective; NA, not applicable.

**Table 2:** The characteristics of studies concentrated on AEs of EV-A71 inactivated vaccine.

Author	Area	Study Period	Age	Vaccine Dose	Male/Female	Type	Main Results
Mao et al. [26]	Wenzhou, China	January 2019–December 2019	6–35 months	29.440	1.27	P	The incidence of AEs in children vaccinated with EV-A71 vaccine was 1.29‰, among which the incidence of local and systemic reactions were 0.95‰ and 0.88‰, respectively. The incidence of AEs in grade I, II, III, and IV were 0.71‰, 0.51‰, 0.07‰, and 0.00‰, respectively
Shen et al. [30]	Zhejiang, China	September 2016–December 2017	6–59 months	32,230	1.07	P	The incidence of AEs within 3 days was 2.045% (one dose) and 1.611 (two dose) respectively. The systemic AEs was high, with an incidence of 1.837% (one dose) and 1.453 (two dose), respectively.
Luo et al. [31]	Zhejiang, China	April 2016–March 2018	6–59 months	71,663	1.08	P	The incidence of AEs within 30 min, 3 d, and 4–30 d were 0.33%, 1.58%, and 0.34%, respectively, and most of the AEs were mild, and most of them were common general reactions, without rare AEs.

Note: d, day; R, retrospective; P, prospective.

**Table 3:** The characteristics of studies concentrated on epidemiological characteristics of HFMD after the introduction of EV-A71 inactivated vaccine.

Author	Study Period	Group Description	Sample	Area	Main Results
Duan et al. [32]	2017–2022	post-vaccination	5115	Chengdu, China	A total of 4.3% presented with severe symptoms, and 4.1% of severe cases experienced significant complications. EV-A71 was no longer the major serotype for laboratory-confirmed HFMD, responsible for 15.6% of severe cases and 1.2% of mild cases.
Meng et al. [33]	2016–2017	post-vaccination	40,000	Xiangyang, China	CV-A6 was the predominant serotype; CVA6 and EV-A71 had proportions of 59.54% and 3.03%, respectively.
Wang et al. [34]	2017–2020	post-vaccination	32,754	Kunming, China	Other enteroviruses replaced EV-A71, and the incidence of EV-A71 decreased dramatically, whereas CV-A6 and CV-A16 had substantial outbreaks in 2018 and 2019, respectively.
Jiang et al [35].	2008–2016 2017–2019	pre-vaccination; post-vaccination	400,704 277,731	Yunnan, China	After the introduction of EV-A71 vaccines, the overall incidence of HFMD increased and reached over 200 cases per 100,000 population-years in 2018 and 2019. However, the case severity and case fatality rate decreased and remained lower than 1 and 0.005% after 2016, respectively. EV-A71-associated mild, severe, and fatal cases sharply decreased.
Huang et al. [36]	2012–2016 2017–2020	pre-vaccination; post-vaccination	82,944 60,436	Hefei, China	The morbidity decreased from 215.22/105 in 2012–2016 to 179.81/105 in 2017–2020 The main pathogenic enterovirus gradually changed from EV-A71 to other enteroviruses, especially CV-A6 after the implementation of EV-A71 vaccination.
Wang et al. [37]	2013–2015 2017–2019	pre-vaccination; post-vaccination	749,736 632,276	Guangxi, China	The proportion of HFMD cases aged 0–12 months decreased from 23.0% to 15.3% between 2013–2015 and 2017–2019; EV-A71 among laboratory-confirmed severe cases in 2013–2015 (62.8%) transformed to other EVs (67.2%) in 2017–2019.
Hong et al. [38]	2013–2015 2017–2019	pre-vaccination; post-vaccination	NR	CDC Reported, China	After the launch of the EV-A71 vaccine, the median age of HFMD patients infected with EV-A71 increased from2.24 years (IQR: 1.43, 3.56) to 2.81 years (IQR:1.58, 4.01). The proportion of patients less than 3 years of age decreased while the proportion of patients 3–5 years of age increased.
Wu et al [39].	2010–2016 2017–2021	pre-vaccination; post-vaccination	NR	Zhejiang, China	The expected incidence will be 2.76 times (include the cases of 2020) and 2.43 times (exclude the cases of 2020) higher than the actual value assuming that the measures of vaccination are not taken. EV-A71 vaccines are very effective and should be administered in the age window between 5 months and 5 years.
Head et al. [40]	2011–2015 2017–2018	pre-vaccination; post-vaccination	134,760 98,698	CDC Reported, China	The average incidence rate of EV-A71 HFMD in 2017–2018 was 60% (95% prediction interval [PI], 41–72%) lower than predicted in the absence of immunization, corresponding to an estimated 6911 (95% PI, 3246–11 542) EV-A71 cases averted over 2 years. There were 52% (95% PI, 42–60%) fewer severe HFMD cases than predicted.

Note: NR, not reported.

## Data Availability

All the data analyzed in this study are available in publicly available databases.

## Supplementary Materials

The following supporting information can be downloaded at https://www.mdpi.com/article/10.3390/vaccines12091028/s1, Table S1: PRISMA_2020_checklist; Table S2: NOS scores of included studies.
